# Undetectable haptoglobin is associated with major adverse kidney events in critically ill burn patients

**DOI:** 10.1186/s13054-017-1837-4

**Published:** 2017-09-26

**Authors:** François Dépret, Chloé Dunyach, Christian De Tymowski, Maïté Chaussard, Aurélien Bataille, Axelle Ferry, Nabila Moreno, Alexandru Cupaciu, Sabri Soussi, Mourad Benyamina, Alexandre Mebazaa, Kevin Serror, Marc Chaouat, Jean-Pierre Garnier, Romain Pirracchio, Matthieu Legrand, for the PRONOBURN group

**Affiliations:** 1 Department of Anesthesiology and Critical Care and Burn Unit, Groupe Hospitalier St-Louis-Lariboisière, Assistance publique – Hôpitaux de Paris, Paris, France; 2 Université Paris Diderot, Paris, France; 3 Service de Biochimie, Hôpital universitaire St-Louis, 1 avenue Claude Vellefaux, Paris, 75010 France; 4 UMR INSERM 942, Institut National de la Santé et de la Recherche Médicale (INSERM), French Clinical Research Infrastructure Network (F-CRIN) Investigation Network Initiative-Cardiovascular and Renal Clinical Trialists (INI-CRCT network), Paris, France; 5 Hôpital Européen Georges Pompidou, 20 rue Leblanc, Paris, 75015 France; 6 Service de Biostatistique et Informatique Médicale, INSERM UMR-1153, Equipe ECSTRA, Hôpital Saint Louis, Sorbonne Paris Cité, Paris, France; 7 French Clinical Research Infrastructure Network (F-CRIN) Investigation Network Initiative-Cardiovascular and Renal Clinical Trialists (INI-CRCT network), Paris, France

**Keywords:** Haptoglobin, Intravascular haemolysis, Acute kidney injury, Burn patients, Major adverse kidney event

## Abstract

**Background:**

Intravascular haemolysis has been associated with acute kidney injury (AKI) in different clinical settings (cardiac surgery, sickle cell disease). Haemolysis occurs frequently in critically ill burn patients. The aim of this study was to assess the predictive value of haptoglobin at admission to predict major adverse kidney events (MAKE) and AKI in critically ill burn patients.

**Methods:**

We conducted a retrospective, single-centre cohort study in a burn critical care unit in a tertiary centre, including all consecutive severely burned patients (total burned body surface > 20% and/or shock and/or mechanical ventilation at admission) from January 2012 to April 2017 with a plasmatic haptoglobin dosage at admission.

**Results:**

A total of 130 patients were included in the analysis. Their mean age was 49 (34–62) years, their median total body surface area burned was 29% (15–51%) and the intensive care unit (ICU) mortality was 25%. Early haemolysis was defined as an undetectable plasmatic haptoglobin at admission. We used logistic regression to identify MAKE and AKI risk factors. In multivariate analysis, undetectable haptoglobin was associated with MAKE and AKI (respectively, OR 6.33, 95% CI 2.34–16.45, *p* < 0.001; OR 8.32, 95% CI 2.86–26.40, *p* < 0.001).

**Conclusions:**

Undetectable plasmatic haptoglobin at ICU admission is an independent risk factor for MAKE and AKI in critically ill burn patients. This study provides a rationale for biomarker-guided therapy using haptoglobin in critically ill burn patients.

## Background

Acute kidney injury (AKI) during an intensive care unit (ICU) stay is associated with increased mortality and morbidity [1, 2]. The pathophysiology of AKI in critically ill patients remains poorly understood. Preventive or curative strategies for AKI are lacking and urgently needed. In the specific population of critically ill burn patients, the prevalence of acute AKI has been reported to be as high as 53%, with mortality rates ranging from 35% to 70% [3–5]. Although haemodynamic alterations, including hypovolaemic shock and low cardiac output, may precipitate the development of AKI [6], other factors are likely to participate [7].

Among the factors contributing to AKI in critically ill burn patients, intravascular haemolysis is a potential candidate. Severe burns have been associated with haemolysis at the early stage of injury [8, 9]. Haemolysis has also been described as a strong causal factor for AKI in other situations such as sickle cell disease [10, 11] or after cardiopulmonary bypass for cardiovascular surgery [12, 13]. The pathophysiology of renal toxicity is complex and multifactorial, involving (1) cell-free haemoglobin (fHb), which is a scavenger of nitric oxide (NO), thereby decreasing its bioavailability and inducing systemic vasoconstriction; and (2) a direct toxicity of fHb that aggregates into casts in the tubular lumen [14, 15]. Better and earlier identification of haemolysis-related AKI may allow the selection of patients who could benefit from specific and innovative treatments such as intravenous haptoglobin administration [16]. The objective of this study was to evaluate the association between plasma haptoglobin level—a widely available biomarker of intravascular haemolysis—and occurrence of major adverse kidney events (MAKE) and AKI in critically ill burn patients.

## Methods

### Study design and population

We conducted a single-centre cohort study in the burn unit of St. Louis Hospital (Assistance Publique - Hôpitaux de Paris), Paris, France. The study was approved by our local ethics committee (PRONOBURN study, comité de protection des personnes IV, St-Louis Hospital; Institutional Review Board 00003835, protocol 2013/17NICB). All medical records of the patients admitted to our intensive care burn unit between January 2012 and April 2017 were screened. All burn patients meeting the following criteria were included in the study: total body surface area (TBSA) burned > 20%, and/or mechanical ventilation at admission, and/or catecholamine infusion at admission, and a measurement of haptoglobin upon admission.

### Outcomes

The primary endpoint of the study was MAKE at day 90. The secondary endpoints were AKI and death within 90 days. MAKE was defined as a composite of the following criteria: death within 90 days, new renal replacement therapy (RRT) during ICU stay, and/or no renal recovery (defined as a ratio of serum creatinine at ICU discharge to serum creatinine at admission > 125%) [17]. AKI was defined and staged according to the Kidney Disease: Improving Global Outcomes criteria [17]. Serum creatinine (Screat) at hospital admission was used to define the baseline Screat.

### Measurements

We collected the following data: age, sex, body mass index, TBSA, full-thickness body surface area burned, mechanism of injury and patient characteristics, Simplified Acute Physiology Score II (SAPS II), Abbreviated Burn Severity Index (ABSI) [18], Unit Burn Standard [19], treatments administered during the first 7 days after admission (hydroxocobalamin, aminoglycosides, vasopressors), and 28- and 90-day mortality. Haptoglobin measurement was performed using a haptoglobin assay (Hitachi modular P analyser; Roche, Paris, France) that is based on the principle of immunological agglutination. Anti-haptoglobin antibodies react with antigen in the sample to form antigen-antibody complexes, which, after agglutination, can be determined turbidimetrically. The reference values of haptoglobin are from 0.3 g/L to 2 g/L; the detection limit is 0.1 g/L; and the linearity limit is 5.2 g/L.

### Patient management

Patients were resuscitated according to the St. Louis Hospital Intensive Care Burn Unit resuscitation protocol with the following haemodynamic targets: mean arterial blood pressure > 65 mmHg, 0.5 ml/kg/h less than urine output < 1 ml/kg/h, 2.5 L/minute/m^2^ less than cardiac index < 3 L/minute/m^2^ and central venous oxygen saturation > 70%. Norepinephrine was administered when required (diastolic arterial blood pressure < 50 mmHg and/or systemic vascular resistance index < 1250 dyn/second/cm^−5^/m^2^). Patients received initial fluid resuscitation using an intravenous bolus of Ringer’s lactate 0.25 ml/kg/%TBSA/h (which corresponds to the 2 ml/kg/%TBSA in the first 8 h of the Parkland formula) with fluid infusion adjusted to reach pre-defined haemodynamic targets. Cardiac function was systematically assessed on admission by echocardiography. Cardiac index was measured by transpulmonary thermodilution using a PiCCO2 monitor (Pulsion Medical Systems AG, Munich, Germany). The PiCCO monitor was calibrated every 2 h during the first 48 h.

Albumin 20% was administered to patients with TBSA > 30% after the sixth hour after thermal injury to reach a serum albumin concentration of 30–35 g/L. When mechanical ventilation was initiated, tidal volume was limited to 6–7 ml/kg to maintain an inspiratory plateau pressure < 30 cmH_2_O and a transpulmonary driving pressure < 15 cmH_2_O. Early enteral nutrition was initiated within 24 h of admission. Glycaemic control was adjusted to maintain glucose levels between 5 and 9 mmol/L. Surgical treatment included escharotomy or fasciotomy as needed and early coverage of excised burn wounds with autografts and/or allografts within the first 7 days after admission.

### Statistical analysis

Continuous variables are reported as mean and SD or median (25th–75th percentile range) as appropriate. Categorical variables are expressed as count (percent). Categorical variables were compared using the chi-square test or Fisher’s exact test as appropriate. Continuous variables were compared using Student’s *t* test or the Mann- Whitney *U* test as appropriate.

Variables associated with MAKE and AKI in univariate analysis were entered in a multivariable logistic regression model with Lasso penalization [20] to identify the factors independently associated with the outcome. Inference was obtained using the post-selection inference method for *L*_1_-penalized models described by Taylor and Tibshirani [21]. Considering the rule of thumb suggesting at least five to ten events for each predictor variable included in the model [22], only the most clinically relevant were included in the multivariate model: creatinine at admission, SAPS II, ABSI, undetectable haptoglobin, need for catecholamine during the first 7 days, and administration of hydroxocobalamin. Model performance was estimated using the cross-validated (tenfold) AUC. Mortality during the first 90 days was described using the Kaplan-Meier estimate and modelled using a Cox proportional hazards model. Survival curves were compared using the log-rank test. In all comparisons, a *p* value < 0.05 was considered statistically significant. All analyses were performed using R software version 3.3.3 for Mac (R Foundation for Statistical Computing, Vienna, Austria).

## Results

### Study population

**Fig. 1:**
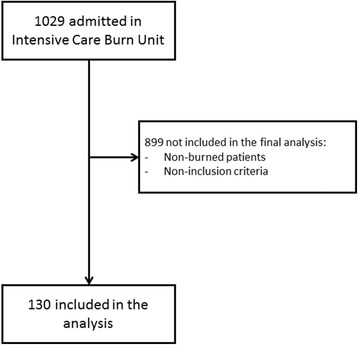
Flowchart

Between January 2012 and April 2017, 1029 patients were admitted (Fig. 1). Of these, 899 did not meet the inclusion criteria. The characteristics of the 130 patients included in the study are summarised in Table 1.

**Fig. 2:**
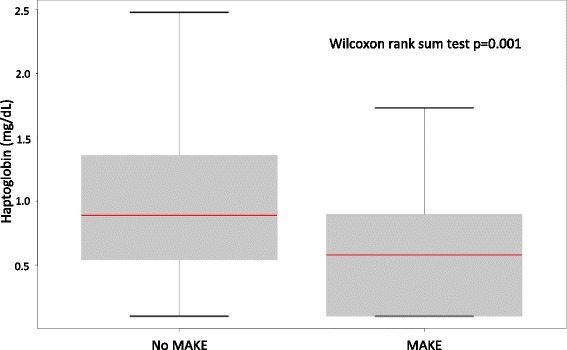
Box plot displaying minimal haptoglobin level between major adverse kidney events (MAKE) and no MAKE

**Table 1:** Patient characteristics

	All patients (*n* = 130)	MAKE (*n* = 41)	No MAKE (*n* = 89)	*p* Value
Age, years	48.5 (34–61.8)	53 (47–67)	44 (29–58)	0.02
Sex, *n* (%)				
Male	80 (61.5)	24 (58.5)	56 (62.9)	0.7
Female	50 (38.5)	17 (41.5)	33 (37.1)	
BMI, kg/m^2^	25.1 (22.7–28.5)	26.2 (22.8–30.6)	24.9 (22.7–27.5)	0.06
Co-morbidities, *n* (%)				
Hypertension	27 (20.8)	13 (31.7)	14 (15.7)	0.06
Diabetes mellitus	16 (12.3)	8 (19.5)	8 (9)	0.15
Chronic kidney disease	2 (1.5)	1 (2.4)	1 (1.1)	0.53
Vascular disease	8 (6.2)	3 (7.3)	5 (5.6)	0.71
Obesity	21 (16.1)	12 (29.3)	9 (10.1)	0.01
Smoking	14 (10.8)	2 (4.9)	12 (13.5)	0.22
Alcohol consumption	15 (11.5)	5 (12.2)	10 (11.2)	1
Psychiatric	17 (13.1)	6 (14.6)	11 (12.4)	0.78
Burn type, *n* (%)				
Thermal	123 (94.6)	41 (100)	82 (92.1)	0.1
Electrical	7 (5.4)	0 (0)	7 (7.9)	
Body surface area burned (%)				
Total	28.5 (15–50.8)	50 (22.5–70)	20 (12–40)	<0.0001
Full thickness	15 (4–32.2)	25 (15–56)	8 (1.8–25)	<0.0001
ICU length of stay, days	27.5 (10–42.5)	18 (3–38)	29 (13.2–45)	0.42
Death in ICU, *n* (%)	33 (25.4)	33 (80.5)	0 (0)	<0.0001
Day of death	15 (3–32)	15 (3–32)	–	
SAPS II	33 (19–49)	45 (35–72)	24 (15–39)	<0.0001
UBS	67 (24–146)	110 (65–224)	46 (16–113)	<0.0001
ABSI	8 (6–10)	11 (8–12)	7 (5–9)	<0.0001
Serum creatinine on admission, μmol/L	67 (65–113)	88 (67–118)	64 (54–77)	<0.0001
Maximal blood lactate concentration within 7 days, mmol/L	4.3 (2.8–7)	7 (5.2–9.4)	3.2 (2.2–4.7)	<0.0001
Minimal serum haptoglobin level, g/L	0.6 (0.1–1.2)	0.1 (0.1–0.5)	0.8 (0.5–1.4)	<0.0001
Undetectable haptoglobin level, *n* (%)	39 (30)	28 (68.3)	11 (12.4)	<0.0001

*Abbreviations: BMI* Body Mass Index, *ICU* Intensive Care Unit, *SAPS II* Simplified Acute Physiology Score II, *UBS* Unit Burn Standard, *ABSI* Abbreviated Burn Severity Index

Data are expressed as median ± 25th–75th interquartile range for continuous variables and count (percent) for discrete variables

### Outcomes

Forty-one patients developed MAKE, including 33 (80.5%) patients who died within 90 days. Twenty-six patients required RRT, and 25 patients had no renal recovery. Seventy-three patients developed AKI during the first 7 days, including 29 (39.7%) patients with stage 1 AKI, 17 (23.3%) with stage 2 and 27 (37%) with stage 3 AKI (Table 2). Only one patient developed AKI after 7 days. Among the 73 patients who developed AKI during the first 7 days, 32 (44%) died during their ICU stay.

**Table 2:** Renal outcomes

	All patients (*n* = 130)	MAKE (*n* = 41)	No MAKE (*n* = 89)	*p* Value
Kidney aggression factors within 7 days, *n* (%)				
Aminoglycoside	5 (3.9)	5 (12.2)	0 (0)	0.0026
Hydroxocobalamin	24 (18.5)	14 (34.1)	10 (11.2)	0.0031
Shock (use of catecholamine)	58 (44.6)	34 (82.9)	24 (27)	<0.0001
AKI within 7 days, *n* (%)	73 (56.1)	39 (95.1)	34 (38.2)	<0.0001
KDIGO stage of patients with AKI within 7 days	2 (1–3)	3 (2–3)	1 (1–2)	<0.0001
Serum creatinine, μmol/L				
Admission	67 (54–90)	88 (67–118)	64 (54–77)	<0.0001
Maximum within 7 days	80 (65–113)	135 (85–206)	74 (64–86)	<0.0001
ICU discharge (alive, without RRT)	49 (35–63)	64 (43–85)	49 (35–61)	0.07
Oliguria within 7 days, *n* (%)	63 (48.5)	35 (85.4)	28 (31.5)	<0.0001
RRT *n* (%)				
Within 7 days	18 (13.8)	18 (44)	0 (0)	<0.0001
During ICU stay	26 (20)	26 (63.4)	0 (0)	<0.001
Alive at ICU discharge	1 (0)	1 (2.4)	–	

*Abbreviations: AKI* Acute kidney injury, *KDIGO* Kidney Disease Improving Global Outcomes, *ICU* Intensive care unit, *RRT* Renal replacement therapy

Data are expressed as median ± 25th–75th interquartile range for continuous variables and number (percent) for discrete variables

#### Haptoglobin and outcomes

**Fig. 3:**
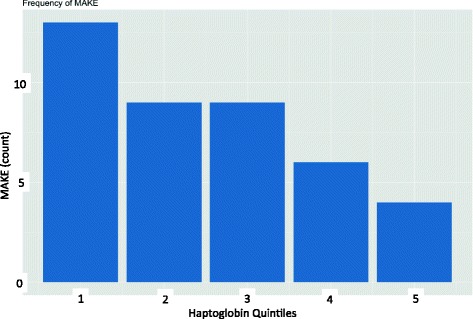
Major adverse kidney event (MAKE) count by haptoglobin quintiles

Undetectable plasma haptoglobin was observed in 39 patients (30%) at ICU admission. All but one had a total burned surface area > 20%. Of these 39 patients, 34 (87.2%) developed AKI and 29 (74.4%) developed MAKE. The minimal haptoglobin concentration was significantly lower in patients with MAKE than in patients without (*p* = 0.002) (Fig. 2). The frequency of MAKE by haptoglobin quintile is illustrated in Fig. 3.

**Fig. 4:**
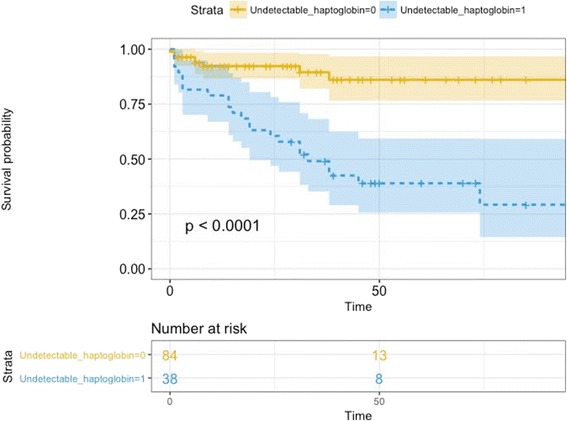
Kaplan-Meier curve between patients with detectable and undetectable haptoglobin in the 72 first h

In univariate analysis, undetectable haptoglobin was strongly associated with MAKE (OR 15.27, 95% CI 6.34–39.66, *p* < 0.001). In multivariate analysis, undetectable haptoglobin (OR 8.32, 95% CI 2.86–26.40, *p* < 0.001), admission creatinine (OR 1.02, 95% CI 1.00–1.03, *p* = 0.05), need for catecholamine during the first 7 days (OR 4.71, 95% CI 1.54–15.49, *p* = 0.01) and SAPS II score (OR 1.04, 95% CI 1.01–1.07, *p* = 0.02) were independently associated with MAKE (Table 3) (model discrimination AUC 0.88, 95% CI 0.80–0.96).

**Table 3:** Multivariable Lasso penalized logistic model: variables associated with major adverse kidney events

Variable	OR	95% CI	*p* Value
Admission creatinine	1.02/μmol/L	1.00–1.03	0.03
SAPS II	1.03/1 point	1.00–1.06	0.05
ABSI	1.14/1 point	0.88–1.35	0.19
Shock in the first 7 days	4.18	1.44–10.59	0.02
Haptoglobin undetectable	6.33	2.34–16.45	<0.001

*SAPS II* Simplified Acute Physiology Score II, *ABSI* Abbreviated Burn Severity Index

After adjustment, admission creatinine (OR 1.02, 95% CI 1.00–1.03, *p* = 0.05), SAPS II (OR 1.04, 95% CI 1.01–1.07, *p* = 0.02), shock within 7 days (OR 4.71, 95% CI 1.54–15.49, *p* = 0.01) and undetectable haptoglobin (OR 8.32, 95% CI 2.86–26.40, *p* < 0.001) were independently associated with the occurrence of AKI. In patients with undetectable haptoglobin, the risk of death during the first 90 days was higher (23 [59%] of 39 versus 9 [10%] of 91; *p* < 0.0001) (Fig. 4). This remained true after adjusting on severity (HR 5.11, 95% CI 2.21–11.78, *p* < 0.001).

#### Factors associated with undetectable haptoglobin

In multivariable analysis, TBSA was independently associated with undetectable haptoglobin (OR 1.04, 95% CI 1.00–1.07, *p* = 0.03), whereas ABSI, smoke inhalation and SAPS II were not (respectively, OR 1.16, 95% CI 0.89–1.5, *p* = 0.27; OR 1.88, 95% CI 0.72–4.95, *p* = 0.20; and OR 1.01, 95% CI 0.98–1.03, *p* = 0.54).

## Discussion

We observed 30% of undetectable plasma haptoglobin in our cohort of critically ill burn patients. The main finding of this study is that undetectable haptoglobin on admission after burn injury is strongly and independently associated with the occurrence of MAKE, AKI and 90-day mortality in critically ill burn patients.

The association between haemolysis and AKI after major aortic surgery [12] and in sickle cell disease [11] has already been described. This is the first study, to our knowledge, describing the association between haptoglobin level and MAKE or AKI in a population of critically ill burn patients. In 1943, Shen et al. [8] described a 25% incidence of haemolysis in 40 patients with combined second-degree and third-degree thermal burns over 15–65% of the body area. Eleven patients developed haemolysis as evidenced by the presence of haemoglobinuria. In the literature on thermal burns, haemolysis is infrequently reported as a complication of severe third-degree burns [8]. In the present study, 38 (>97%) of 39 cases with undetectable haemolysis had > 20% TBSA. TBSA was strongly associated with undetectable haptoglobin, even after adjustment for confounding factors. Physiopathology of renal toxicity of haemolysis is probably multifactorial, including (1) the role of the fHb azote monoxide (NO) scavenger, decreasing its bioavailability and therefore inducing systemic vasoconstriction; and (2) direct toxicity of fHb aggregating into casts in the tubular lumen [14, 15].

We describe a high incidence of AKI in the first week (56%), which is close to the incidence reported by Palmieri et al., who described an incidence of 53% in a retrospective cohort of 60 severely burned patients (>20% TBSA) [3]. In their study, they did not describe the prevalence of MAKE; however, AKI was strongly associated with mortality because 34% of patients with AKI died, whereas no deaths were reported among patients without AKI. In the present study, we chose to use MAKE as the primary endpoint [17]. Of note, MAKE were driven largely by RRT and mortality in our cohort. Moreover, the prognosis of patients needing RRT was very poor, with a 90-day mortality of 78%, which is again in accord with the available literature. Yoon et al. recently reported 84% mortality in burn patients receiving RRT [23].

Recently, in a single-centre, retrospective, observational Japanese study, intra-operative administration of haptoglobin was independently associated with a lower risk of AKI after cardiovascular surgery [16], suggesting a protective role of haptoglobin in binding fHB and therefore preventing its potential toxicity on the kidney. As far as we know, the use of haptoglobin in burn patients has been described only in a case report [24] of a 24-year-old man with 100% TBSA and in a series of five patients [25]. In the case report, haptoglobin was administered three times over the first 24 h after the detection of haemoglobinuria. Despite the severity of the initial burn, the patient did not develop AKI in the five 5 days, suggesting a protective effect of intravenous haptoglobin administration [24].

Our study has some limitations. First, this was an observational study, and thus it describes an association between haemolysis, MAKE and AKI, but not necessarily a causal relationship. Second, it was a single-centre study. This may limit the generalisability of the results. However, our results rely on a strong pathophysiological background and may open a window for establishing methods to prevent MAKE and AKI in this population. Third, low haptoglobin level could arise from conditions other than haemolysis. However, the differential diagnoses appear to be very unlikely in our population. Haptoglobin was measured upon admission, before hepatic dysfunction occurs in critically ill patients. Finally, even though burn patients are frequently transfused after surgery for excision, none were transfused in the early phase (first 96 h), and none had associated trauma or haemorrhage.

## Conclusions

This study shows that undetectable haptoglobin was strongly associated with the development of MAKE, AKI and 90-day mortality in a large cohort of patients with severe burns. Further multicentre studies should be performed to confirm these results. Interventional studies using recombinant haptoglobin should be considered in the future to improve outcome in patients with severe burns.

## Abbreviations

ABSI: Abbreviated Burn Severity Index
AKI: Acute kidney injury
BMI: Body mass index
fHb: Cell-free haemoglobin
ICU: Intensive care unit
KDIGO: Kidney Disease: Improving Global Outcomes
MAKE: Major adverse kidney event
RRT: Renal replacement therapy
SAPS II: Simplified Acute Physiology Score II
Screat: Serum creatinine
TBSA: Total body surface area
UBS: Unit Burn Standard
